# Lactoferrin Adsorbed onto Biomimetic Hydroxyapatite Nanocrystals Controlling - *In Vivo* - the *Helicobacter pylori* Infection

**DOI:** 10.1371/journal.pone.0158646

**Published:** 2016-07-06

**Authors:** Andrea Fulgione, Nunzia Nocerino, Marco Iannaccone, Sante Roperto, Federico Capuano, Norberto Roveri, Marco Lelli, Antonio Crasto, Armando Calogero, Argenia Paola Pilloni, Rosanna Capparelli

**Affiliations:** 1 Department of Agriculture, University of Naples “Federico II”, Portici (Naples), Italy; 2 Department of Veterinary Medicine and Animal Productions, Division of Infectious Diseases, University of Naples “Federico II”, Naples, Italy; 3 Department of Food Inspection, Istituto Zooprofilattico Sperimentale del Mezzogiorno, Portici (Naples), Italy; 4 Department of Chemistry “G. Ciamician”, Alma Mater Studiorum, University of Bologna, LEBSC, Bologna, Italy; 5 Advanced Biomedical Science Department, University of Naples “Federico II", Naples, Italy; 6 Department of Experimental Medicine, Division of Clinical Bacteriology, Second University of Naples, Naples, Italy; University of Helsinki, FINLAND

## Abstract

**Background:**

The resistance of *Helicobacter pylori* to the antibiotic therapy poses the problem to discover new therapeutic approaches. Recently it has been stated that antibacterial, immunomodulatory, and antioxidant properties of lactoferrin are increased when this protein is surface-linked to biomimetic hydroxyapatite nanocrystals.

**Objective:**

Based on these knowledge, the aim of the study was to investigate the efficacy of lactoferrin delivered by biomimetic hydroxyapatite nanoparticles with cell free supernatant from probiotic *Lactobacillus paracasei* as an alternative therapy against *Helicobacter pylori* infection.

**Methods:**

Antibacterial and antinflammatory properties, humoral antibody induction, histopathological analysis and absence of side effects were evaluated in both in vitro and in vivo studies.

**Results:**

The tests carried out have been demonstrated better performance of lactoferrin delivered by biomimetic hydroxyapatite nanoparticles combined with cell free supernatant from probiotic *Lactobacillus paracasei* compared to both lactoferrin and probiotic alone or pooled.

**Conclusion:**

These findings indicate the effectiveness and safety of our proposed therapy as alternative treatment for *Helicobacter pylori* infection.

## Introduction

During its coevolution with the host, *Helicobacter pylori* acquired the capacity to grow in the stomach, a particularly harsh niche. Humans represent the prevalent natural host of this pathogen. In the stomach, *Helicobacter pylori* can assume either the replicating or dormant forms [1] and can cause different gastric pathologies (peptic ulcer, chronic gastritis) representing the major risk factor for gastric carcinoma [2]. It is transmitted by the oral or oro-fecal routes [3] and colonizes about 50% of the human population [2]. *Helicobacter pylori* infection is more frequent in developing countries where contaminated water, high population density or not adequate personal hygiene favor the transmission of the pathogen [4].

The standard triple therapy for *H*. *pylori* treatment, is based on the use of clarithromycin, proton pump inhibitor (PPI) plus amoxicillin or metronidazole [5]. However, the spread of *H*. *pylori* clarithromycin resistance strains, caused a significant decline in the efficacy of this standard regimen[6]. For these reasons, new therapeutic approaches are needed. Recent studies have shown the use of probiotics—as adjuvant—in the *H*. *pylori* therapy, improve the eradication rates by reducing bacterial adhesion or colonization [7,8] while at the same time, the side effects are decreased. Moreover, it has been reported that biological properties of lactoferrin (LF) are improved when this protein is surface-linked to biomimetic hydroxyapatite (HA) nanocrystals. In particular, the adsorption of lactoferrin onto hydroxyapatite significantly improved the antimicrobial, antioxidant and immunomodulatory activities of the native protein and the bioactive surface of HA functionalized with LF could be utilized to improve the material performance towards the biological environment for biomedical applications [9]. In addition, the HA does not affect appreciably the conformation of the LF after the adsorption process; in fact, using FT-Raman and FT-IR, the protein adsorbed resulted slightly unfolded with a small fraction of the α-helix structure converted into turn, while the β-sheet content remained almost unaltered [10].

Here, we have evaluated—*in vitro* and *in vivo—*a new therapy against *H*. *pylori* infection exploiting the increased biological properties of lactoferrin adsorbed onto biomimetic hydroxyapatite (LF-HA) and combined with cell free supernatant (CFS) from *Lactobacillus paracasei*. Indeed, lactoferrin in addition to its main biological function as iron transporter, shows also increased antioxidant, antitumor, antimicrobial and immunomodulatory properties when functionalized on biomimetic HA nanocrystals [9–13]. *Lactobacillus paracasei* is already used in the therapy against *Helicobacter pylori* to contrast diarrhea caused by prolonged use of antibiotics [14]. In addition, *Lactobacillus paracasei* is also known to favorably influence the immune response of the host [15], acting locally (the gastrointestinal tract) and at distant sites [16]. Thus, results obtained in this study, strongly suggest the use of LF-HA and CFS from *Lactobacillus paracasei* as alternative to conventional antibiotic treatment.

## Materials and Methods

### Ethics Statement

All animal protocols were approved by Ethical Animal Care and Use Committee of University of Naples Federico II under Protocol number 2012/0133280 approved in data December, 07 2012. Institutional guidelines are in compliance with the EU legislation 86/609/EEC and with National legislative decree n. 116/92. During the experiments with animals, all efforts were made to minimize suffering.

### Biomimetic HA Nanocrystals

Biomimetic HA nanocrystals were synthesized as previously reported [17], with a carbonate content of 5 ±2%, resembling that of bone HA nanocrystals where the carbonate content ranges from 4 wt% to 8 wt%. The HA nanocrystals have been synthesized in order to obtain crystals with chemical physic characteristics very close to those previously described [9]. Biomimetic HA nanocrystals were precipitated from an aqueous solution of Ca (OH)_2_ 0.17 M by slow addition of an aqueous solution of H_3_PO_4_ 0.15 M. Synthesized HA nanocrystals exhibit a calcium deficiency as a result of surface disorder resembling bone HA nanocrystals.

### Preparation of LF-Coated HA

Synthetic biomimetic HA nanocrystals were surface-functionalized at pH 7.4 by different amounts of lactoferrin molecules using the previously described [10]. The increase in LF concentration in the buffer solution enhances the HA nanocrystals surface coverage until it is complete. Isotherm LF adsorption onto biomimetic HA nanocrystals where the adsorbed amount (C Lactoferrin, in mg/m^2^) is plotted against the protein concentration after adsorption (C Lactoferrin in mg/mL) has been utilized to evaluate the amount of LF surface immobilization on HA at pH 7.4.

### Electron Microscopy

Transmission electron microscopy investigations were carried out using a 1200 EX microscope fitted with link elemental dispersive X-ray analysis detectors and a 3010 UHR operating at 300 kV (JEOL Ltd, Tokyo, Japan). The powdered samples were ultrasonically dispersed in ultrapure water and a few droplets of the slurry were then deposited on perforated carbon foils supported on conventional copper microgrids. Scanning electron microscopy observations were carried out using an 840A microscope (JEOL Ltd). The specimens were mounted on aluminum stubs using carbon tape and covered with a coating of Au-Pd approximately 10 nm thick using a coating unit (Polaron Sputter Coater E5100, Polaron Equipment, Watford, UK).

### X-Ray Diffraction Analysis

X-ray diffraction powder patterns were collected using Analytical X’Pert Pro equipped with an X’Celerator detector powder diffractometer with Cu Ka radiation generated at 40 kV and 40 mA.

### Determination of Specific Surface Area

Measurements were done using a Sorpty 1750 instrument (Carlo Erba) using N2 absorption at 77 K and the well known Brunauer, Emmett, and Teller procedure [18].

### Bacteria

*Helicobacter pylori* (HP) type strain ATCC 43504 (or NCTC 11637), used in this study, was obtained from American Type Culture Collection (USA). The identity of the pathogen was confirmed by PCR assay of *H*. *pylori* specific gene *glm*M [19]. HP was grown in 10 ml of liquid brain heart infusion medium (BHI; Oxoid, UK) supplemented with 10% (w/v) fetal bovine serum (FBS; Oxoid, UK), and incubated under microaerophilic conditions generated by the CampyGen system (Oxoid) at 37°C, as described [20]. For *in vitro* and *in vivo* studies, bacteria were harvested in exponential phase (OD600 nm; 0-6-0.8) by centrifugation (3500 g for 5 min), and suspended in Mueller Hinton broth (MH, Oxoid, UK) for antimicrobial activity or in sterile NaCl 0,9% to infect mice respectively. The probiotic *Lactobacillus paracasei* was obtained from by the Department of Microbiology of the University of Naples Federico II. Cell free supernatant (CFS) of *Lactobacillus paracasei* was obtained as described previously [21–22]. The supernatant was tested to identify the presence of bacteria by plating serial dilutions on MRS Agar (Oxoid England). No bacteria were identified after filtration [21–22].

### *In Vitro* Measurement of Antibacterial Activity

*In vitro* assay was carried out as previously described [23]. Briefly, HP (10^6^ CFU/ml) was incubated overnight with: lactoferrin (LF; 200–600 μg/ml); lactoferrin adsorbed on nanoparticles of hydroxyapatite (LFH; 200–600 μg/ml); CFS from *Lactobacillus paracasei* (P; 50 μl/ml); lactoferrin (200–600 μg/ml) plus CFS from *Lactobacillus paracasei* (50 μl/ml) (LFP); lactoferrin adsorbed on nanoparticles of hydroxyapatite (200–600 μg/ml) plus CFS from *Lactobacillus paracasei* (50 μl/ml) (LFHP); conventional antibiotic pool (amoxicillin 200–600 μg/ml and clarithromycin 200–600 μg/ml; AP). The minimal concentration of the antimicrobial at which 100% inhibition of growth (MIC100) was determined by measuring the absorbance at 600 nm (Biorad microplate reader model 680, Hercules, CA) as reported [20].

### *In Vivo* Experiments

Three groups of twelve-week-old Balb/C mice (12 animals/group) were orally infected with HP (10^6^ CFU/mouse in 100 μl PBS). One more group of mice was treated orally with PBS (100 μl/mouse). At 3 weeks post infection, feces were collected and tested by PCR to detect the presence of HP. The PCR test was carried out as described [19]. At this stage, two groups of mice received at weekly intervals: three doses of lactoferrin adsorbed on nanoparticles of hydroxyapatite plus CFS from *Lactobacillus paracasei* (LFHP; 300 μg/mouse and 50 μl/mouse); three doses of the antibiotic pool (AP; amoxicillin 300 μg/mouse plus clarithromycin 300 μg/mouse). Within each group, 3 mice were sacrificed at 1, 2 and 3 weeks after treatment. Feces and blood samples, in addition to the stomach, were collected and stored at -80°C till tested.

### Cytokines Measurement

The levels of the cytokines TNF-α, IFN-γ, IL-17, IL-4, IL6, IL-10, IL-12 and COX-2 present in stomachs homogenates were determined by Elisa as described [24] using antibodies from R&D.

### Other Methods

Histological examinations of stomachs were carried out by standard hematoxylin-eosin stain on specimen fixed in 10% formalin.

IgG (in the serum) and IgA (in stomachs homogenates) were measured by immunological assay [25] using Mouse *Helicobacter pylori* IgG (MBS725427, MyBioSource Inc, San Diego CA) and IgA (MBS745410, MyBioSource Inc, San Diego CA) ELISA Kit. Leukocytes and hemochrome were determined with the Am45 instrument (Melet Schloesing, Osny, France). All the results were analyzed by the Student’s t test and only p<0.05 were considered significant.

## Results

### Biomimetic HA Nanocrystals

**Fig 1:**
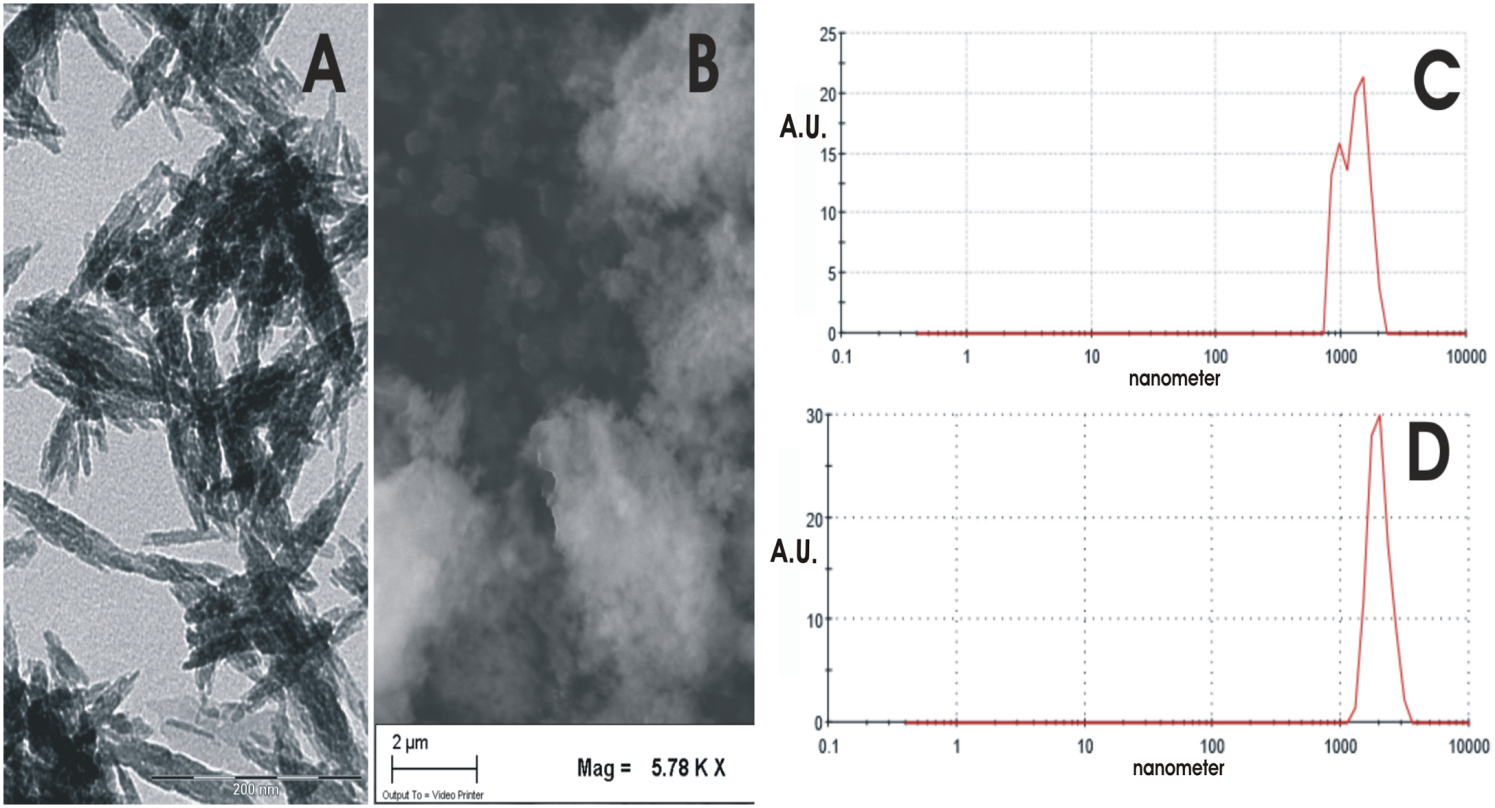
Electron microscopy pictures. A) Transmission electron microscopy (TEM) image of the biomimetic hydroxyapatite nanocrystal synthesized with a lamellar acicular morphology mimicking the bone biogenic hydroxyapatite nanocrystals. B) Scanning electron microscopy (SEM) image of the biomimetic hydroxyapatite nanocrystals aggregated in micrometrics clusters. C) DLS plot of the synthesized nanocrystals revealing that they are aggregate in micrometric clusters nanostructured about 1.3±0.3μm large. D) DLS plot of the nanohybrid composites made of biomimetic HA nanocrystals surface covered by Lactoferrin which aggregate spontaneously in clusters about 1,9 μm large.

Hydroxyapatite nanocrystals have been synthesized with composition, structure, morphology, dimension and surface reactivity very close to bone nanocrystals. Biomimetic HA nanocrystals have been synthesized as previously reported [17] with a carbonate content of 5% ± 2%. The presence of few units % of phosphate anions substituted by carbonate anions produces a pseudo amorphous layer without crystalline order on the surface of the nanocrystals. In fact for these nanocrystals, a surface calcium/phosphorus molar ratio of 1:3 can be determined by X-ray photoemission spectroscopy (XPS) analysis against a nearly stoichiometric calcium/phosphorus molar ratio of 1:7 when determined in bulk by inductively coupled plasma (ICP) analysis indicating calcium deficiency as a result of the surface disorder resembling bone HA nanocrystals [26]. This surface amorphous layer is responsible for the zeta potential of -20.5±1.5 mV shown by the HA nanocrystals at physiological pH (7.4). The powder X-ray diffraction pattern of the synthesized nanocrystals indicates a relatively low degree of crystallinity, about 40–45% if calculated according to the method described by Sherman [27]. This value is close, but higher respect to the value of about 30% determined by the X-ray diffraction pattern for natural deproteinated bone nanocrystals. This finding together with the high specific surface area of about 110 m^2^/g, which is only slightly lower than the value of 120 m^2^/g obtained for biogenic bone nanocrystals is essential for the high surface reactivity of these biomimetic. The lamellar morphology of these nanocrystals (length and width about 110±5 nm and 20±3 nm, respectively, and a thickness of about 8±2 nm) mimics bone hydroxyapatite. In Fig 1A the transmission electron microscopy (TEM) image of a synthetic hydroxyapatite nanocrystal is reported together in Fig 1C, with the DLS profile obtained for the synthesized hydroxyapatite revealing that nanocrystals aggregate spontaneously in clusters about 1,3 μm large. Scanning electron microscopy (SEM) image of the biomimetic hydroxyapatite nanocrystals spontaneously aggregated in micrometrics clusters is reported in Fig 1B.

### Preparation of Lactoferrin Coated Biomimetic HA Nanocrystals

Synthetic biomimetic HA nanocrystals were surface-functionalized at pH 7.4 by different amounts of lactoferrin molecules using the method reported by Iafisco et al [10].

**Fig 2:**
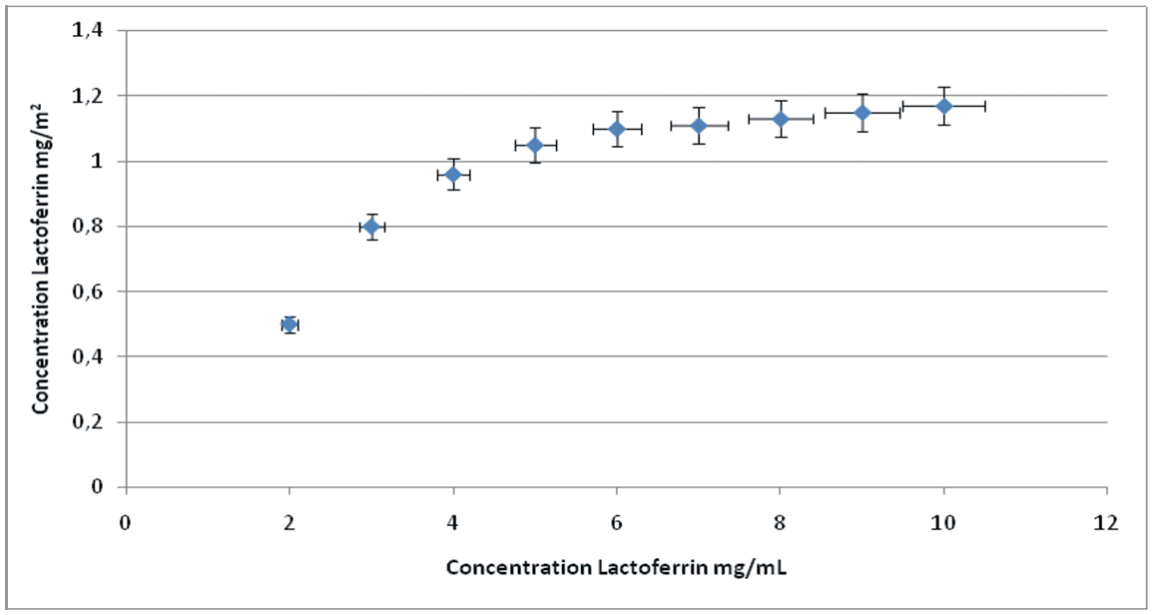
Isotherm adsorption of lactoferrin on hydroxyapatite nanocrystal nanocrystals. Adsorption isotherm of lactoferrin on biomimetic hydroxyapatite nanocrystals at pH 7.4. The adsorbed lactoferrin is plotted against the protein concentration after adsorption.

Isotherm of lactoferrin adsorption onto biomimetic HA nanocrystals at pH 7.4 is reported in Fig 2, where the adsorbed amount (C Lactoferrin, in mg/m^2^) is plotted against the protein concentration after adsorption (C Lactoferrin in mg/mL). The plot is characterized by an initial slope, indicating high protein affinity for the biomimetic HA surface.

The increase of lactoferrin concentration in the buffer solution enhances the surface coverage until it is complete. The absorption-saturation yields a plateau value corresponding to the maximum amount of lactoferrin surface immobilization of about 0.8 mg/m^2^. The isoelectric point of lactoferrin is 8.5, and it thus has a net positive charge below the isoelectric point [28]. At pH 7.4, the positive electrostatic surface potential of lactoferrin produces a strong surface interaction, with the negative HA nanocrystals (zeta potential of -20.5±1.5 mV) avoiding protein–protein interaction. This electrostatic interaction leads to formation of a lactoferrin monolayer coated onto the HA nanocrystals. The HA morphology and the nanodimension do not appreciably affect the conformation of the absorbed lactoferrin. At pH 7.4, the lactoferrin covering the HA nanocrystals appeared to be only slightly unfolded, with a small fraction of the alpha-helix structure being converted into turn while the beta-sheet content remained almost unchanged [10]. Nanohybrid composites made of biomimetic HA nanocrystals surface covered by lactoferrin of 0.8 mg/m^2^ (LFH) exhibit a surface area of about 90 m^2^/g, appreciably smaller than the 110 m^2^/g of the synthesized biomimetic HA nanocrystals. This finding agrees with the fig 1C and 1D. In fact, while the fig 1C shows the low dimension of nanostructured micrometric aggregates are 1,3 μm, in the Fig 1D it is possible to observe the enlargement of the LF-HA aggregates up to 1,9 μm. Additional informations about the chemical characterization are reported in S1 File.

LFHP composites have been prepared in order to show by *in vitro* and *in vivo* tests its efficacy to control *Helicobacter pylori* infection respect conventional antibiotic treatment.

### *In Vitro* Evaluation of Therapies

**Fig 3:**
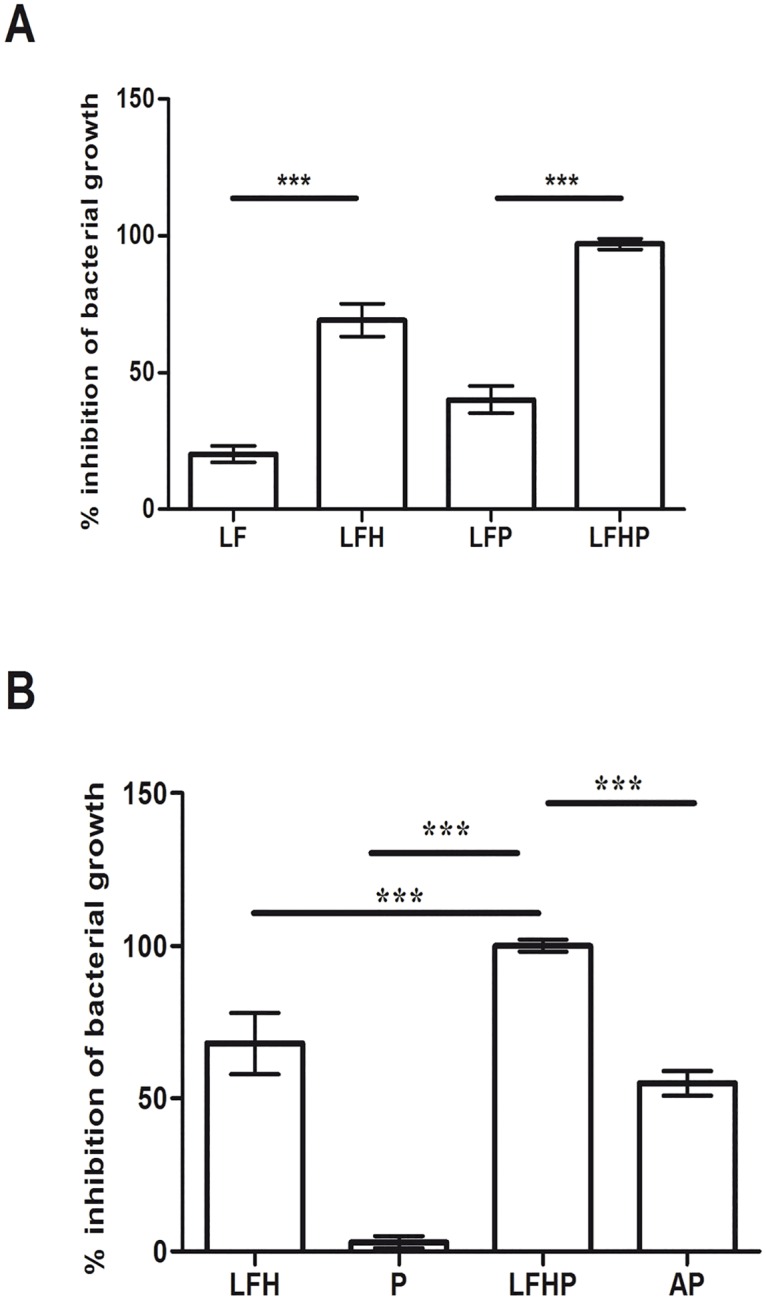
Modulation of antimicrobial effects against *Helicobacter pylori*. Antimicrobial activity against *Helicobacter pylori* (10^6^ CFU/ml) displayed by: A) (LF) lactoferrin (300μg/ml); (LFH) lactoferrin adsorbed on nanoparticles of hydroxyapatite (300 μg/ml); (LFP) lactoferrin plus *cell free* supernatant (CFS) from *Lactobacillus paracasei* (300 μg/ml and 50 μl/ml); (LFHP) lactoferrin adsorbed on nanoparticles of hydroxyapatite plus CFS from *Lactobacillus paracasei* (300 μg/ml and 50 μl/ml). B) (LFH) lactoferrin adsorbed on nanoparticles of hydroxyapatite (300 μg/ml); (P) *CFS* (50 μl /well); (LFHP) lactoferrin adsorbed on nanoparticles of hydroxyapatite plus CFS from *Lactobacillus paracasei* (300 μg/ml and 50 μl /ml); (AP) antibiotic pool (amoxicillin 300 μg/well and clarithromycin 300 μg/ml). Results are presented as mean value ± S.D and are representative of three independent experiments, each performed in triplicate. *** p value<0.001.

*Helicobacter pylori* (10^6^ CFU/ml) was incubated overnight with lactoferrin alone (LF) or LF in combination with cell free supernatant (CFS) from *Lactobacillus paracasei* (LFP therapy). The results displayed that *Lactobacillus paracasei* supernatant enhances the antimicrobial activity of LF (Fig 3A). Next, we explored the efficacy of synthetic hydroxyapatite nanoparticles, which have no significant antibacterial and anti-inflammatory activities (data not show), in combination with LF and CFS from *Lactobacillus paracasei* (LFHP therapy). LFHP displayed a higher antimicrobial activity compared to LF or LFP (Fig 3A and 3B). The results were confirmed by the measurement of diameters of inhibition zones on solid plate (data not shown). The activity of a drug molecule without interaction with HA is usually lower respect the activity of the same drug molecule after interaction with the HA nanocrystal surface [29]. This important finding is due to the HA nanocrystals large surface area (120m^2^/g) which allows to drug molecules to be widely sprayed in the biological environmental increasing their biological activity.

### *In Vivo* Evaluation of Therapies

**Fig 4:**
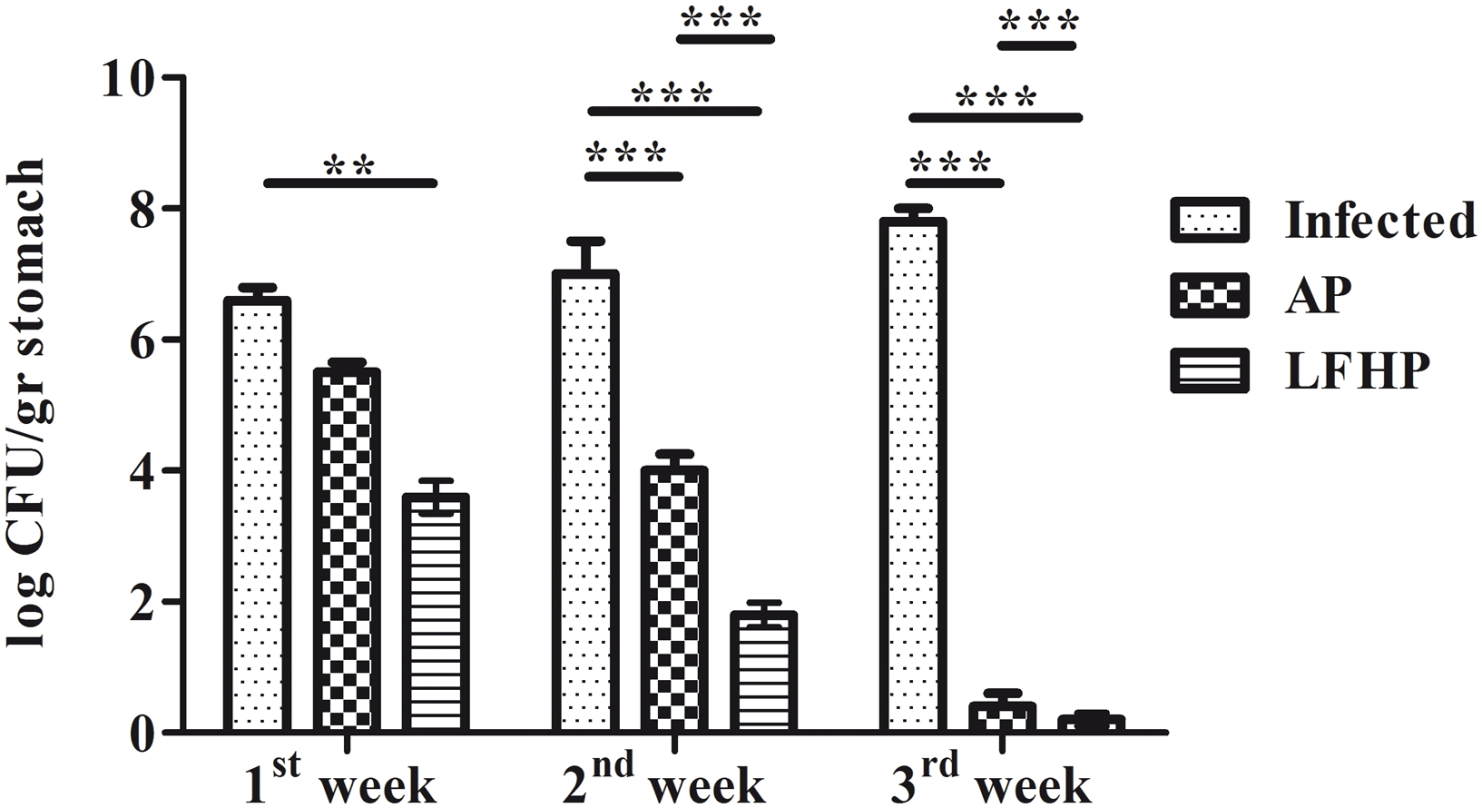
Stomach bacterial load of *H*. *pylori* infected mice. Bacterial load in stomach of: (Infected) mice infected with *Helicobacter pylori* (10^6^ CFU/mouse); (AP) infected with *Helicobacter pylori* (10^6^ CFU/mouse) and treated with antibiotic pool (amoxicillin 300 μg/mouse plus clarithromycin 300 μg/mouse); (LFHP) infected with *Helicobacter pylori* (10^6^ CFU/mouse) and treated with with lactoferrin adsorbed on nanoparticles of hydroxyapatite plus CFS from *Lactobacillus paracasei* (300 μg/mouse plus 50μl /mouse). Data are presented as mean value ± S.D and are representative of three independent experiments, each performed with 6 animals/group. *** p value<0.001.

**Fig 5:**
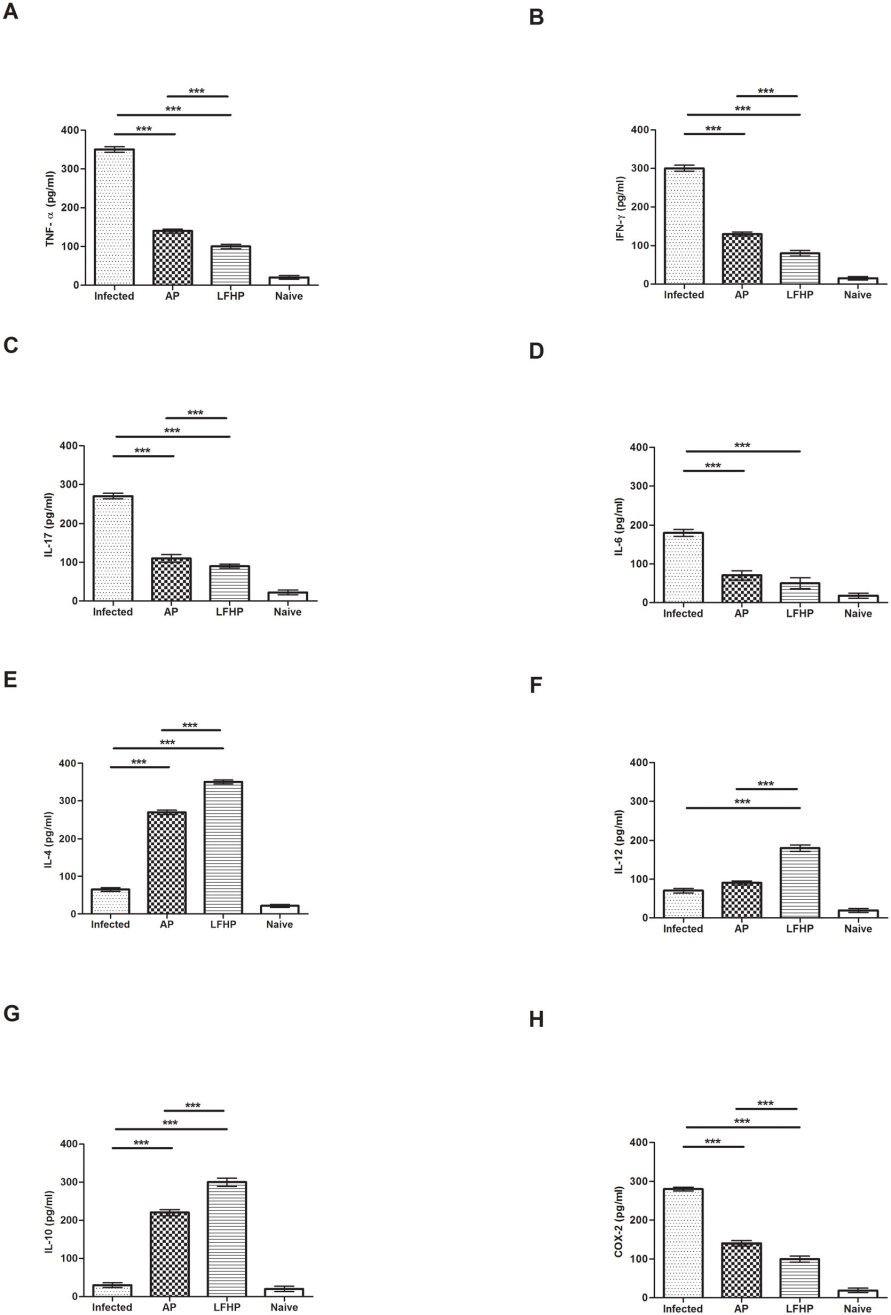
Cytokines immune assay. Immunomodulatory activity is detected by the levels of A) TNF- α, B) IFN- γ, C) IL-17, D) IL-6, E) IL-4, F) IL-12, G) IL-10 and H) COX-2 in: (Infected) mice infected with *Helicobacter pylori* (10^6^ CFU/mouse); (AP) infected with *Helicobacter pylori* (10^6^ CFU/mouse) and treated with antibiotic pool (amoxicillin 300 μg/mouse plus clarithromycin 300 μg/mouse); (LFHP) infected with *Helicobacter pylori* (10^6^ CFU/mouse) and treated with lactoferrin adsorbed on nanoparticles of hydroxyapatite plus CFS from *Lactobacillus paracasei* (300 μg/mouse plus 50μl/mouse); (Naïve) mice neither infected with *Helicobacter pylori* or treated. Results are presented as mean value ± S.D and are representative of three independent experiments, each performed in triplicate. *** p value<0.001.

**Fig 6:**
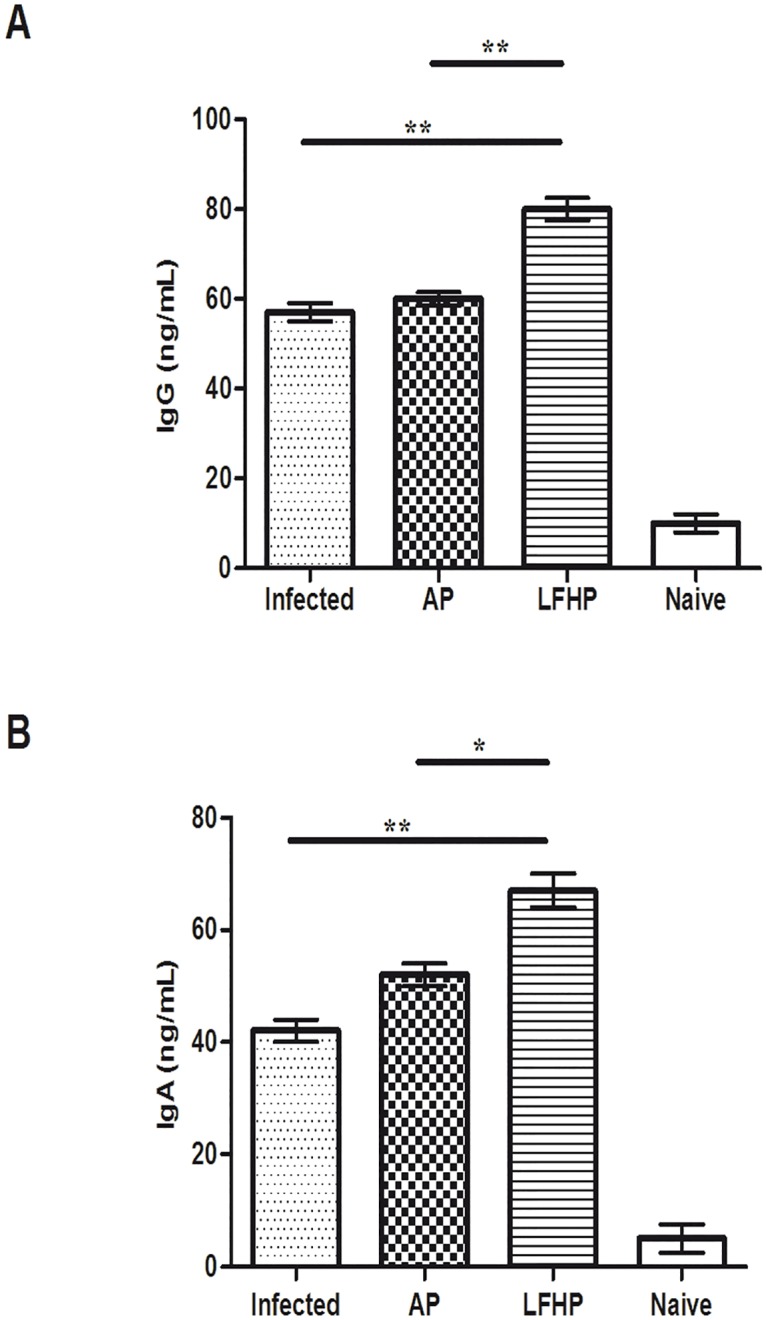
Humoral response in *H*.*pylori* infected mice. Levels of IgG A) in serum and IgA B) in stomach homogenate in: (Infected) mice infected with *Helicobacter pylori* (10^6^ CFU/mouse); (AP) infected with *Helicobacter pylori* (10^6^ CFU/mouse) and treated with antibiotic pool (amoxicillin 300μg/mouse plus clarithromycin 300 μg/mouse); (LFHP) infected with *Helicobacter pylori* (10^6^ CFU/mouse) and treated with lactoferrin adsorbed on nanoparticles of hydroxyapatite plus CFS *Lactobacillus paracasei* (300 μg/mouse plus 50μl /mouse); (Naïve) mice neither infected with *Helicobacter pylori* or treated. The levels were determined by ELISA test and are presented as mean value ± S.D and are representative of three independent experiments, each performed in triplicate. *p value <0.05, ** p value <0.01.

Groups of mice (12 mice/group) were infected orally with *Helicobacter pylori*. After three weeks of post infection, mice were treated with AP or LFHP. Three mice from each group were weekly sacrificed to measure the stomach bacterial load as described [30]. The mice treated with LFHP displayed the lowest load (Fig 4) after the first week while after three weeks of treatment, LFHP effect is similar to AP therapy (Fig 4). These results were confirmed by a quantitative RT-PCR on the feces of *H*. *pylori*-infected mice (S2 Fig). Moreover, LFHP, as AP treatment, displayed a significant anti-inflammatory activity, curbing the production of IFN-γ, TNF-α, IL-17 and COX-2 and inducing also IL-4, IL-10 and IL-12 expression (Fig 5). LFHP also induced a higher humoral response in the blood (IgG) and stomach (IgA) compared to AP (Fig 6) and the specifity against *H*. *pylori* is been verified using Mouse *Helicobacter pylori* IgG (MBS725427, MyBioSource Inc, San Diego CA) and IgA (MBS745410, MyBioSource Inc, San Diego CA) ELISA Kit.

**Fig 7:**
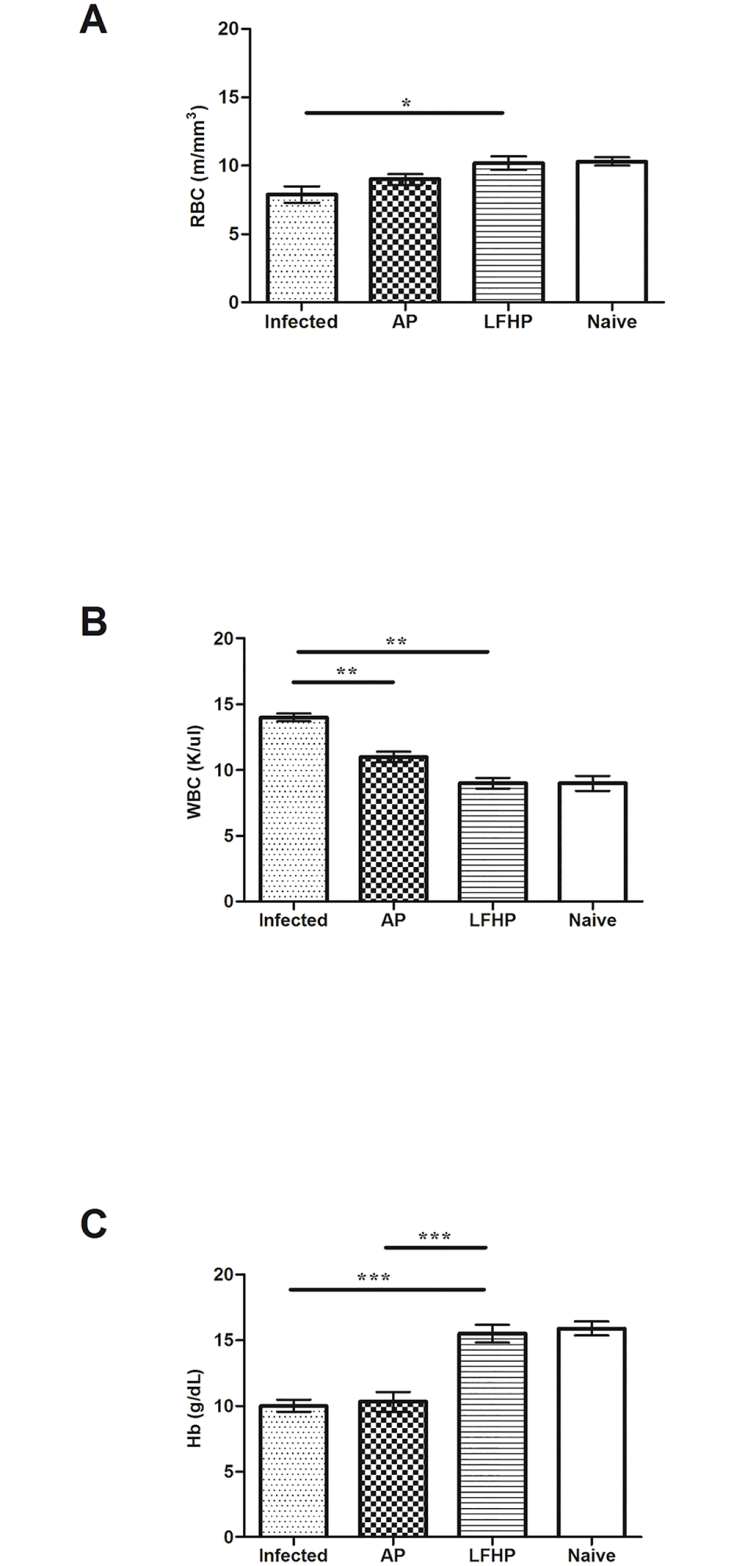
Leucocytes count in *H*. *pylori* infected mice. Levels of A) RBC; B) WBC and C) Hbin blood samples of: (Infected) mice infected with *Helicobacter pylori* (10^6^ CFU/mouse); (AP) infected with *Helicobacter pylori* (10^6^ CFU/mouse) and treated with antibiotic pool (amoxicillin 300μg/mouse plus clarithromycin 300μg/mouse); (LFHP) infected with *Helicobacter pylori* (10^6^ CFU/mouse) and treated with lactoferrin adsorbed on nanoparticles of hydroxyapatite plus CFS from *Lactobacillus paracasei* (300 μg/mouse plus 50μl/mouse); (Naïve) mice neither infected with *Helicobacter pylori* or treated. Results are presented as mean value ± S.D and are representative of three independent experiments, each performed in triplicate. *p value <0.05, ** p value <0.01, *** p value<0.001.

LFHP modulates also blood parameters. In fact, at three weeks post infection, control animals displayed elevated leucocytes level, reduced hemoglobin level and reduced red blood cells numbers while LFHP regulates blood parameters within normal ranges (Fig 7).

**Fig 8:**
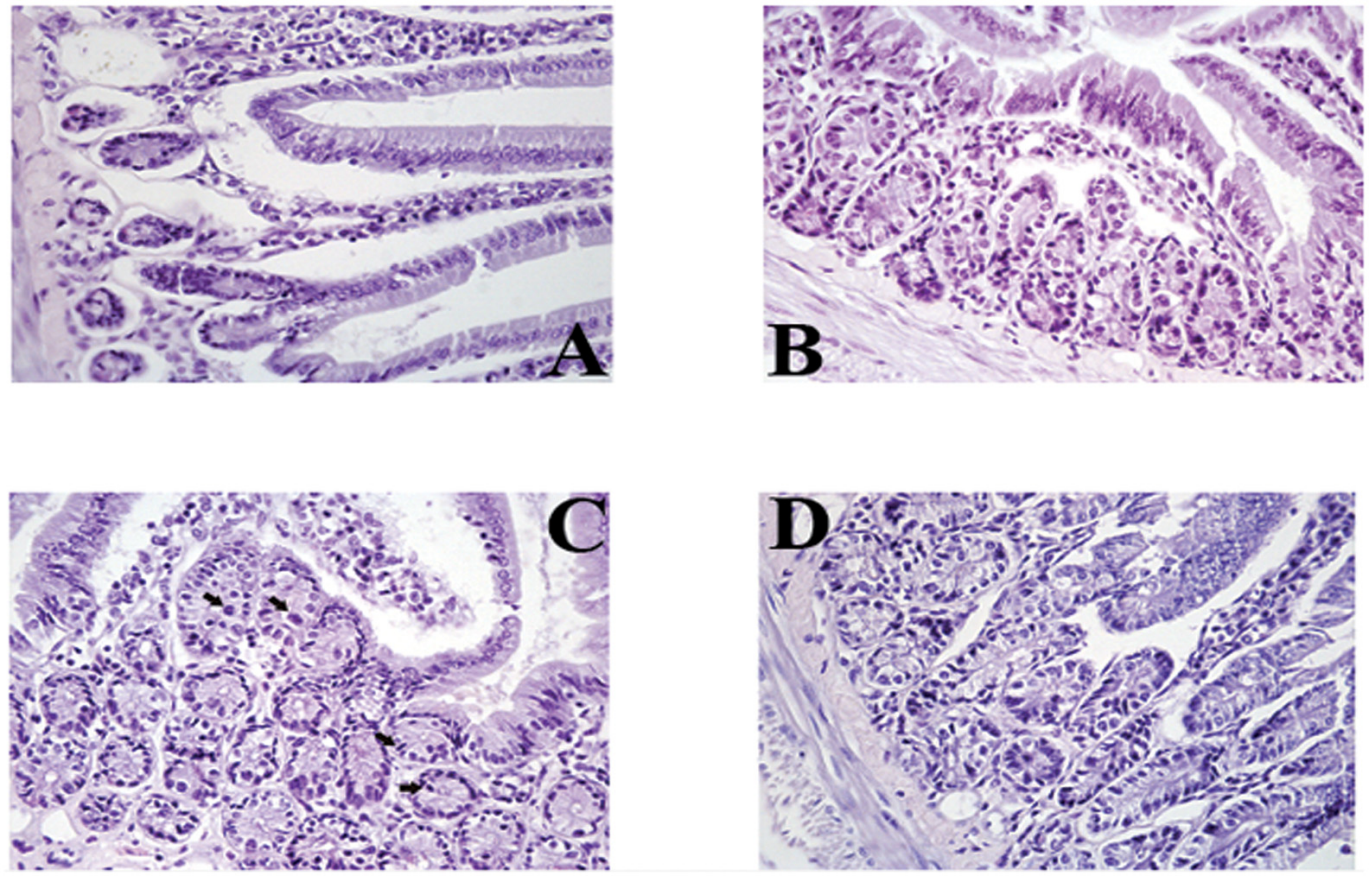
Histology of *H*. *pylori* infected mice. A) *Helicobacter pylori*-infected mouse. The glandular section of gastric mucosa shows severe morphological alteration of gastric mucosa. Notice the heavy infiltration of lamina propria with lymphoid cells. Magnification 40X. B) Glandular section of the gastric mucosa of a mouse infected with *Helicobacter pylori* and then treated with amoxicillin and clarithromycin. Some glandular cells show very mild regressive changes. Magnification 40X; Note the well conserved glandular cells. The microscopic pattern is very similar to that shown in Fig 8D. Magnification 40X. C) Glandular section of the gastric mucosa of a mouse infected with *Helicobacter pylori* and then treated with LFHP. Epithelial cells lining the glandular lumen show normal morphology. Note the presence of numerous mitoses (arrows). Magnification 40X. D) Glandular section of the gastric mucosa of a naive mouse. The morphology of the gastric glands is well conserved. The lamina propria contains few lymphoid cells. The naive mouse does not differ significantly from mice infected with *Helicobacter pylori* and then treated with or LFHP. Magnification 40X.

Histopathological examinations showed differences between mice infected and those treated with LFHP (Fig 8A–8D).

## Discussion

The growing number of *Helicobacter pylori* isolates resistant to conventional antibiotics makes the identification of new molecules active against this pathogen a priority [31]. In this study, we compared a novel antimicrobial treatment with conventional therapy against *Helicobacter pylori*.

Our therapy is based on the use of lactoferrin adsorbed onto hydroxyapatite nanoparticles in combination with the cell free supernatant from *Lactobacillus paracasei*. The antibacterial activity of lactoferrin is attributed to the property of this molecule to sequester iron ions and thus deprive the pathogen of this essential nutrient. In addition, the N-terminal region of the lactoferrin (known as lactoferricin), rich in arginine residues and positively charged, interacts with the negatively charged bacteria. *Lactobacillus paracasei* modulates the gastrointestinal functions of the host [32] and protects the host releasing bacteriocins [33] and metabolites, such as acetic and lactic acids [34].

Preliminary experiments *in vitro* demonstrated that LFHP treatment shows a higher antibacterial activity against *Helicobacter pylori* compared to AP therapy (Fig 3). Based on these results, we evaluated the effect of LFHP treatment in a mouse model. By the third dose, both LFHP and AP controlled the active form of the pathogen (Fig 4 and S2 Fig). However, the presence of *Helicobacter pylori*-specific DNA in the mice infected with *Helicobacter pylori* and then treated with LFHP, suggested that the pathogen could still be present in the host in a coccoid form (data not shown) [1,35].

Moreover, in this study we show that, in addition to TNF-α, LFHP also controls the pro-inflammatory cytokines IFN-γ, IL-17 and IL-10 and induces the production of anti-inflammatory cytokines (Fig 5).

*Helicobacter pylori* infection causes anemia [36], while lactoferrin, administered orally, stimulates antibody response [25]. According to these studies, we observed that mice treated with LFHP display higher levels of antibodies (Fig 6) and free hemoglobin, along with a higher numbers of red blood cells (Fig 7) compared to the mice treated with AP. Thus, LFHP performs better than AP also according to these new parameters (reduced anemia and higher antibody response). The finding that LFHP induces a local (sIgA production at the mucosal level) as well as systemic (IgG production in the blood) immune response is an important property of LFHP. IgG antibodies in fact opsonize bacteria and facilitate phagocytosis and lysosomial activity [25], while sIgA antibodies protect the mucosal surfaces [37]. Histopathological examinations of the stomach show no remarkable differences between the gastric mucosa of LFHP treated mice and uninfected mice; the treatment with LPHP seem consent the regeneration of glandular cells of gastric mucosa,

## Conclusions

In conclusion, we have demonstrated that LFHP therapy offers significant advantages compared to conventional therapy: higher antibacterial, anti-inflammatory and immune response activities and absence of hematological alterations. These results, although preliminary, open new possibilities for the treatment of *Helicobacter pylori* infection.

## Supporting Information

S1 Fig Experimental plan. Flow chart of the experimental design.
(TIF)

S2 Fig Real Time PCR of *Helicobacter pylori* infection. RT-PCR of bacterial detection in the feces of: (Infected) mice infected with *Helicobacter pylori* (10^6^ CFU/mouse); (AP) infected with *Helicobacter pylori* (10^6^ CFU/mouse) and treated with antibiotic pool (amoxicillin 300 μg/mouse plus clarithromycin 300 μg/mouse); (LFHP) infected with *Helicobacter pylori* (10^6^ CFU/mouse) and treated with with lactoferrin adsorbed on nanoparticles of hydroxyapatite plus CFS from *Lactobacillus paracasei* (300 μg/mouse plus 50 μl /mouse). Data are presented as mean value ± S.D and are representative of three independent experiments, each performed with 6 animals/group. *** p value<0.001.
(TIF)

S1 File Chemical characterization of LF-HA. SEM, FT-IR analyses of LF-HA synthesis.
(DOC)
