# Supplementary figures and images for: Lactoferrin Adsorbed onto Biomimetic Hydroxyapatite Nanocrystals Controlling - In Vivo - the Helicobacter pylori Infection
Source: PLoS One. 2016 Jul 6;11(7):e0158646. doi: 10.1371/journal.pone.0158646 (PMC4934871; doi:10.1371/journal.pone.0158646)

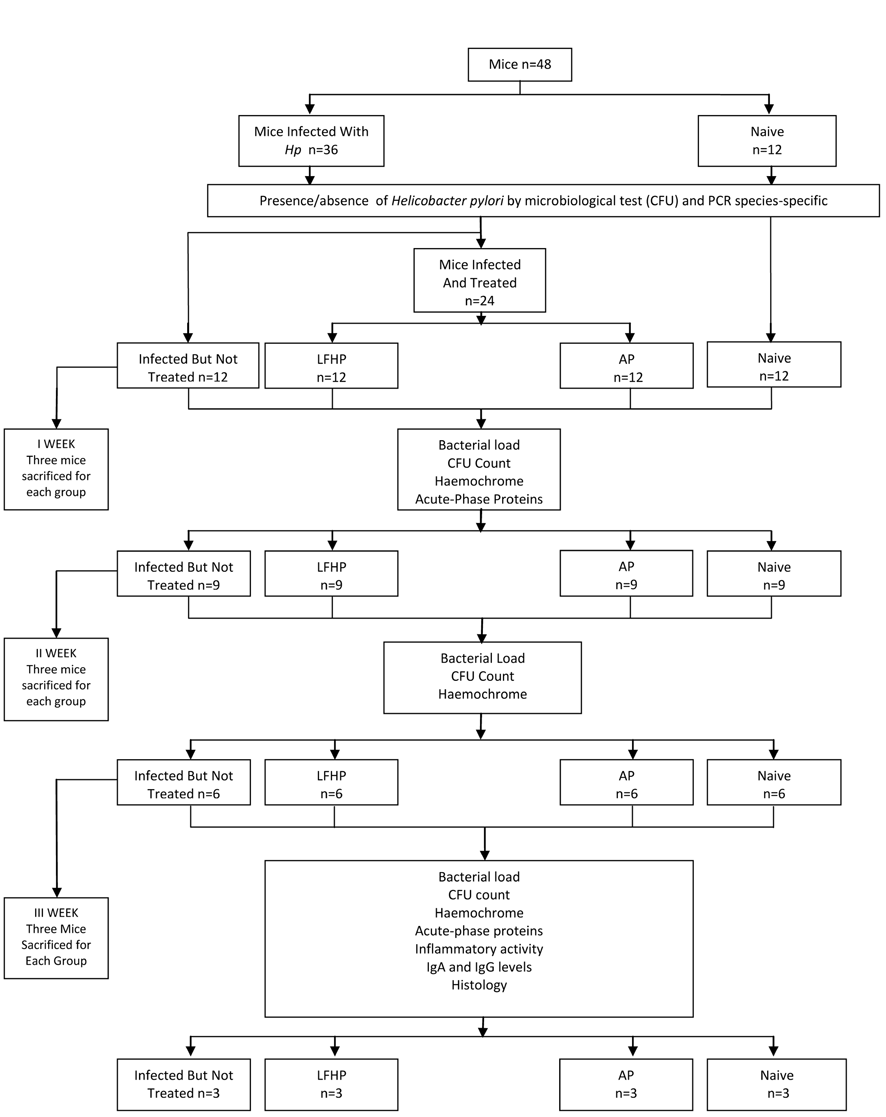

Supplement: S1 Fig — Flow chart of the experimental design. (TIF) [file pone.0158646.s001.tif]

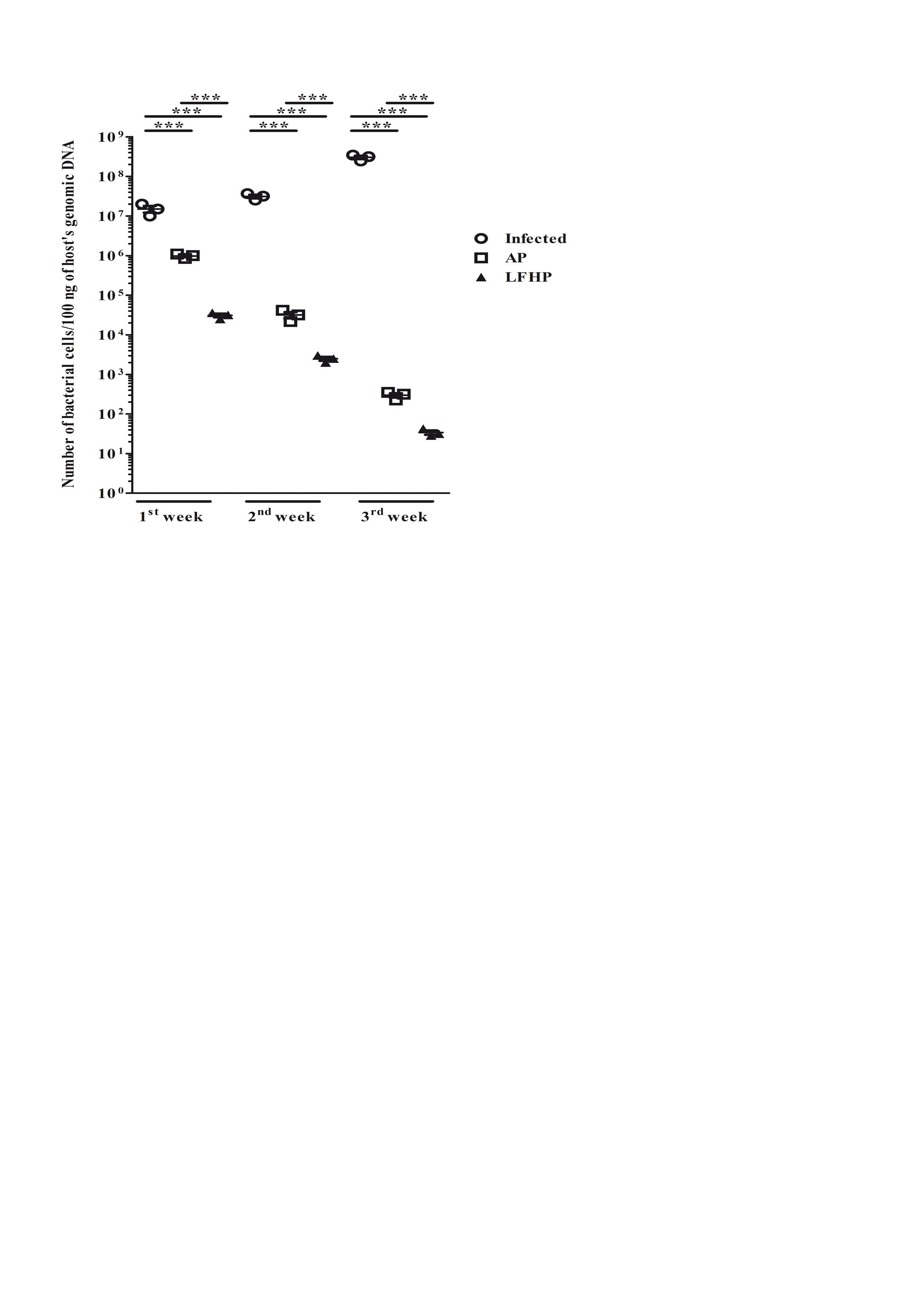

Supplement: S2 Fig — RT-PCR of bacterial detection in the feces of: (Infected) mice infected with Helicobacter pylori (106 CFU/mouse); (AP) infected with Helicobacter pylori (106 CFU/mouse) and treated with antibiotic pool (amoxicillin 300 μg/mouse plus clarithromycin 300 μg/mouse); (LFHP) infected with Helicobacter pylori (106 CFU/mouse) and treated with with lactoferrin adsorbed on nanoparticles of hydroxyapatite plus CFS from Lactobacillus paracasei (300 μg/mouse plus 50 μl /mouse). Data are presented as mean value ± S.D and are representative of three independent experiments, each performed with 6 animals/group. *** p value<0.001. (TIF) [file pone.0158646.s002.tif]
