# Supplementary material for: Lactoferrin Adsorbed onto Biomimetic Hydroxyapatite Nanocrystals Controlling - In Vivo - the Helicobacter pylori Infection
Source: PLoS One. 2016 Jul 6;11(7):e0158646. doi: 10.1371/journal.pone.0158646 (PMC4934871; doi:10.1371/journal.pone.0158646)
Supplement: S1 File — SEM, FT-IR analyses of LF-HA synthesis. (DOC) [file pone.0158646.s003.doc]

The low degree of crystallinity of hydroxyapatite (HA) nanocrystals is very close to the HA natural

bone (Fig 1A-B).

Position [°2Theta]

10

20

30

40

50

Counts

0

400

1600

3600

HA-acetato

**Fig 1A. X-Ray Diffraction of hydroxyapatite nanocrystals**

**Fig 1B. X-Ray Diffraction of bone hydroxyapatite**

In Fig 2A- B are reported the TEM images of HA nanocrystals and HA nanocrystals after the interaction with Lactoferrin (LF), realized following the methods described in the manuscript. The interaction between HA and LF (after the HA synthesis) don’t change the morphology of nanocrystals. The HA morphology, in presence of LF can change only if we add the LF to HA during the HA synthesis (Fig. 2C).


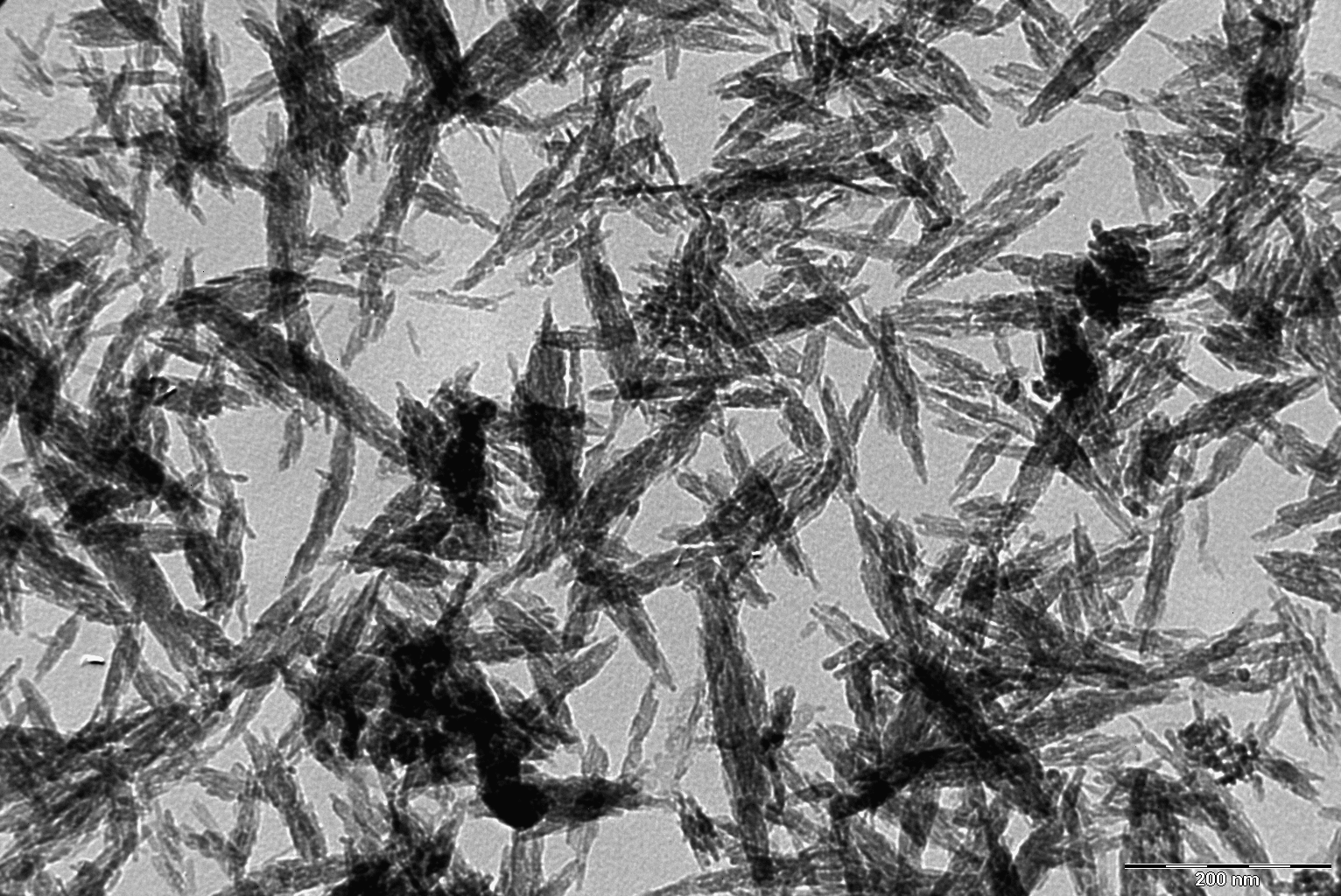


**Fig 2A. TEM image of HA nanocrystals**


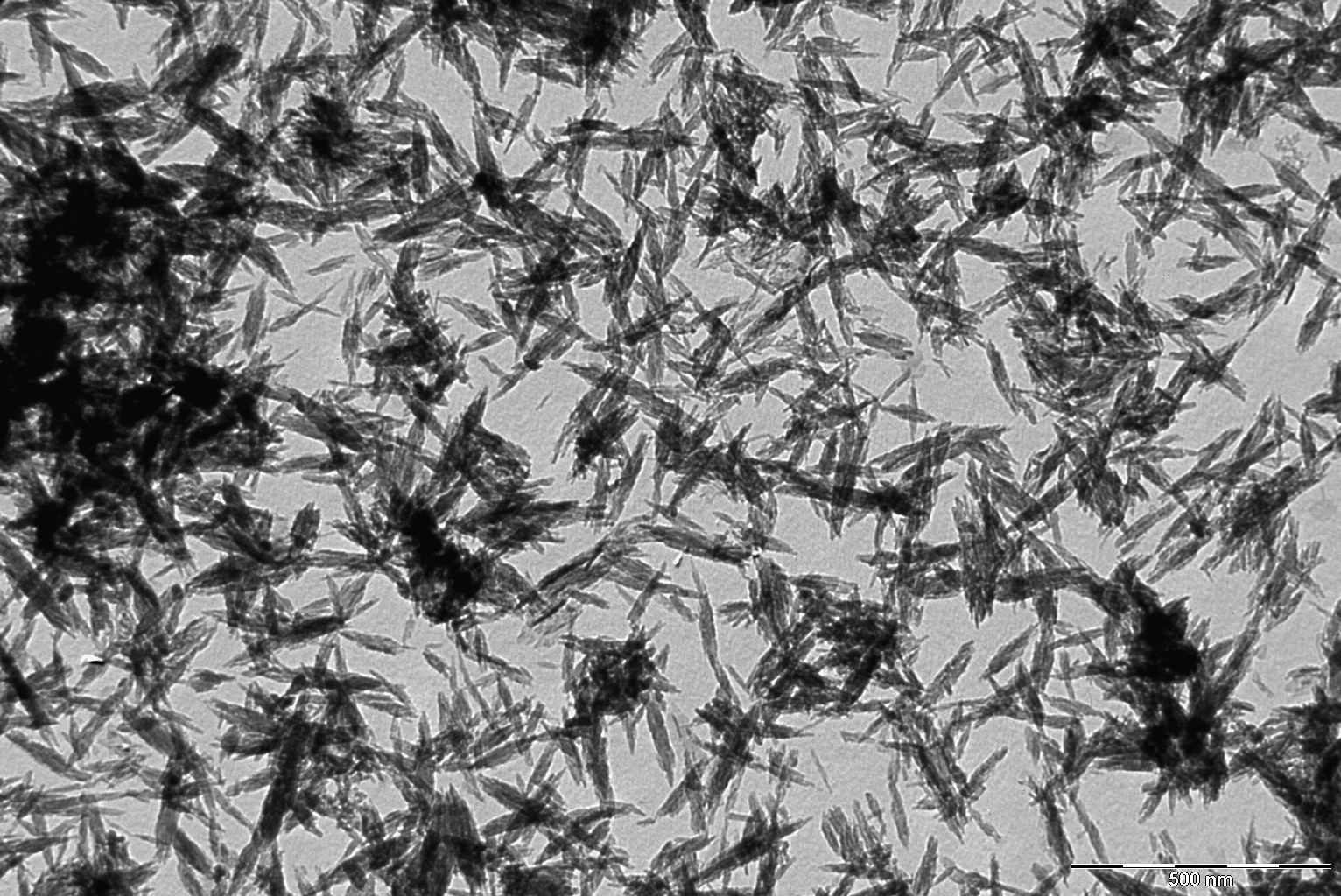


**Fig 2B. TEM image of HA nanocrystals after interaction with LF**

.


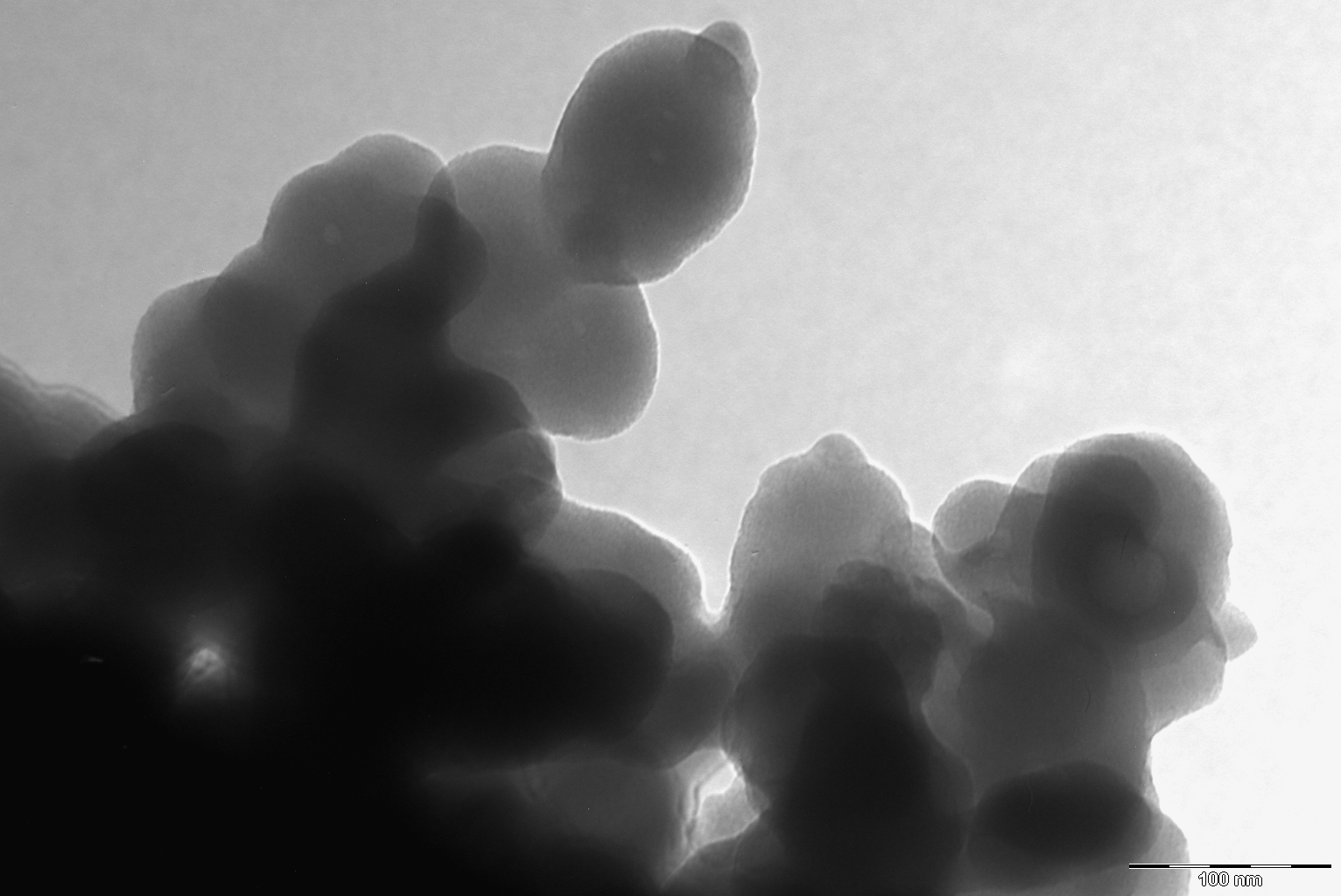


**Fig 2C. TEM image of HA nanocrystals realized inpresence of LF protein**

In order to investigate the structural modifications of the protein adsorbed onto HA nanocrystals, the LF-HA suspension was centrifuged and the precipitate obtained, consisting in protein adsorbed onto surface of HA, was analyzed by FT-IR spectroscopy. Particular attention has been paid on the region of the spectrum between 1800 and 1300 cm^-1^, which is relative to the protein amide I and amide II bands [1-4]. The Fig 3 reported the spectra of lactoferrin and LF-HA molecule. As it shown, the amide I band of lactoferrin adsorbed on HA surface is less enlarged and more centered around 1650 cm^-1^ compared to that of the native protein, pointing out that the the protein was subject to a reduction of its β structures .

**Fig 3. Spectra FT-IR of LF (red spectra) e HA-LF (violet) obtained at pH 7.4.**

**References**

1. Servagent-Noinville S, Revault M, Quiquampoix H, Baron M. Conformational Changes of Bovine Serum Albumin Induced by Adsorption on Different Clay Surfaces: FTIR Analysis. J Colloid Interface Sci. 2000; 221(2):273-283.
2. Carrasquillo KG, Carro JC, Alejandro A, Toro DD, Griebenow K. Reduction of structural perturbations in bovine serum albumin by non-aqueous microencapsulation. J Pharm Pharmacol. 2001; 53(1):115-20.
3. Valerio F, Balducci D, Lazzarotto A. [Adsorption of proteins by chrysotile and crocidolite: role of molecular weight and charge density.](http://www.ncbi.nlm.nih.gov/pubmed/2446862) Environ Res. 1987; 44(2):312-20.
4. Fu K, Griebenow K, Hsieh L, Klibanov AM, LangerR. FTIR characterization of the secondary structure of proteins encapsulated within PLGA microspheres. J Control Release. 1999; 58(3):357-66.
